# Supplementary material for: Functional modulation of primary visual cortex by the superior colliculus in the mouse
Source: Nat Commun. 2018 Sep 25;9:3895. doi: 10.1038/s41467-018-06389-6 (PMC6156231; doi:10.1038/s41467-018-06389-6)
Supplement: Supplementary file 1 — Supplementary Information [file 41467_2018_6389_MOESM1_ESM.pdf]

Supplementary Information

Functional Modulation of Primary Visual Cortex by the Superior Colliculus in the Mouse

Ahmadlou et al.

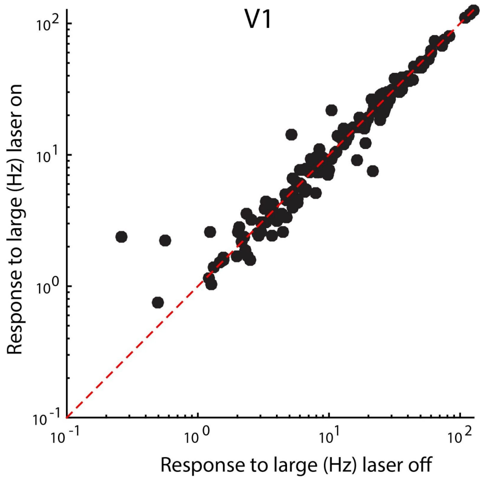

Supplementary Figure 1. No change in V1 response to full screen gratings when sSC was optogenetically inhibited.

Response to full screen gratings in V1 when sSC was optogenetically inhibited by laser versus without laser. Dots represent units ($p = 0.06$, Wilcoxon signed rank test; 14 mice, 160 units).

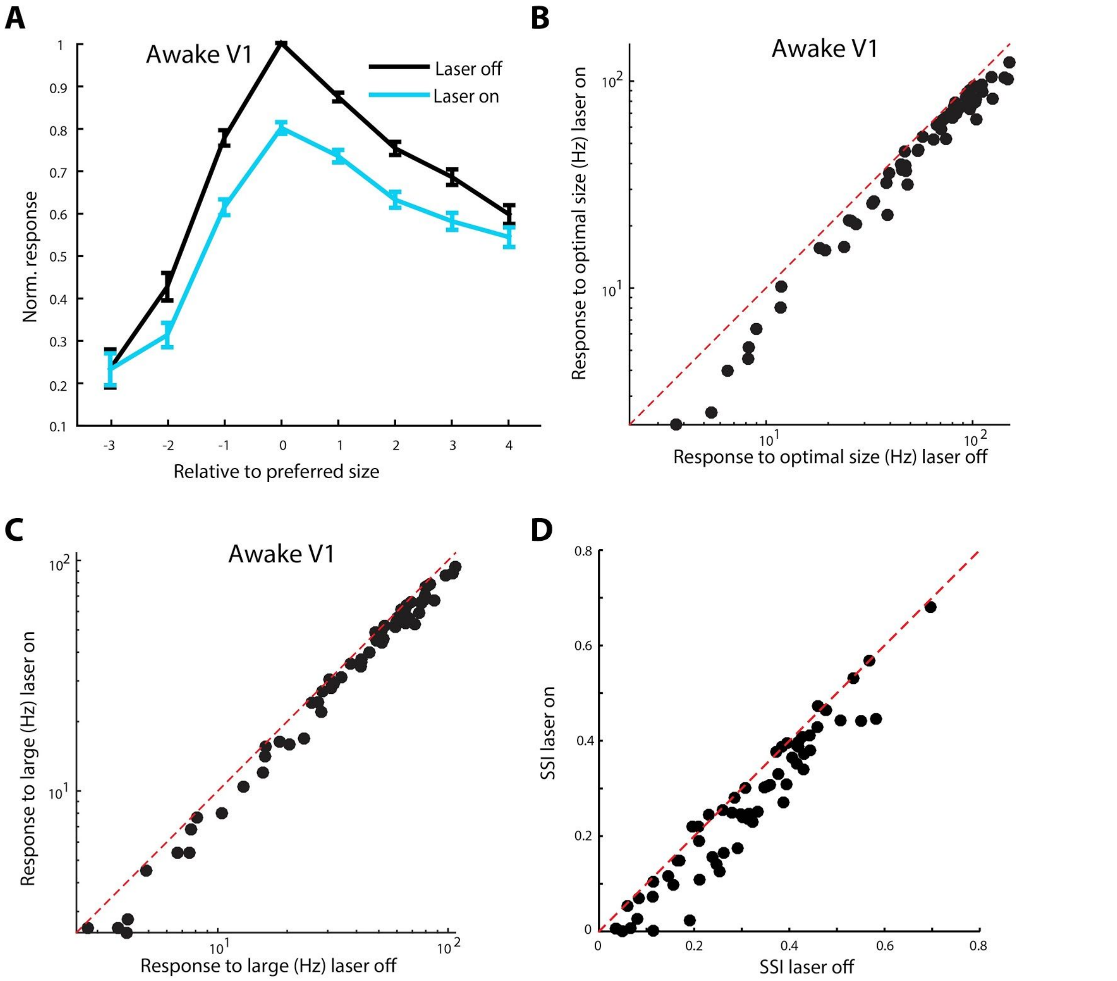

Supplementary Figure 2. Modulation of size tuning in V1 by optogenetically inhibiting sSC in awake mice.

(A) Responses, normalized to response to optimal stimulus without laser light, in V1 of awake mice for different sizes (5 mice, 64 units). The sizes are given as the size step relative to the preferred size. Error bars represent s.e.m.

(B) Response in V1 of awake mice to optimal sized stimuli, with and without laser light on sSC. Dots represent units ($p = 3.5 \times 10^{-12}$, Wilcoxon signed rank test, 5 mice, 64 units).

(C) Response in V1 of awake mice to largest size stimuli, with and without laser light on sSC ($p = 4.1 \times 10^{-12}$, Wilcoxon signed rank test).

(D) Surround suppression index (SSI) in V1 of awake mice, with and without laser light on sSC ($p = 1.4 \times 10^{-12}$, Wilcoxon signed rank test)

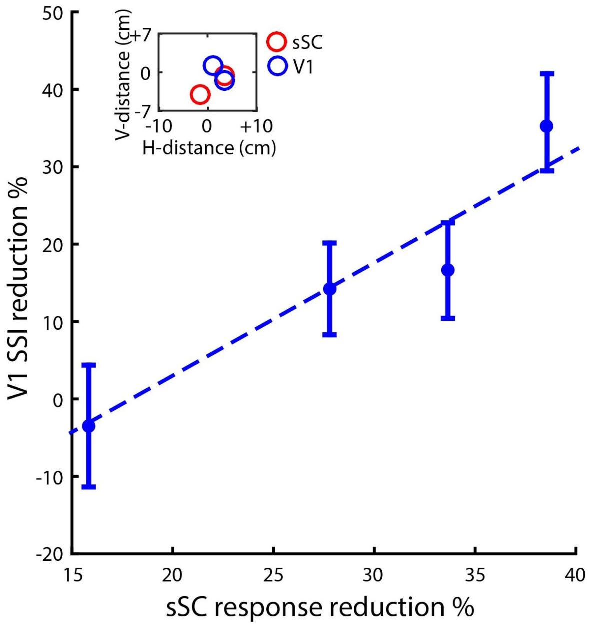

Supplementary Figure 3. V1 SSI reduction correlated with level of sSC silencing.

The amount of activity reduction in the sSC when the laser was turned on at four different levels was strongly correlated to the induced reduction in V1 SSI (Pearson r = 0.95, p = 0.0004, non-zero slope test; 1 mouse, 13 units). Error bars indicate s.e.m.

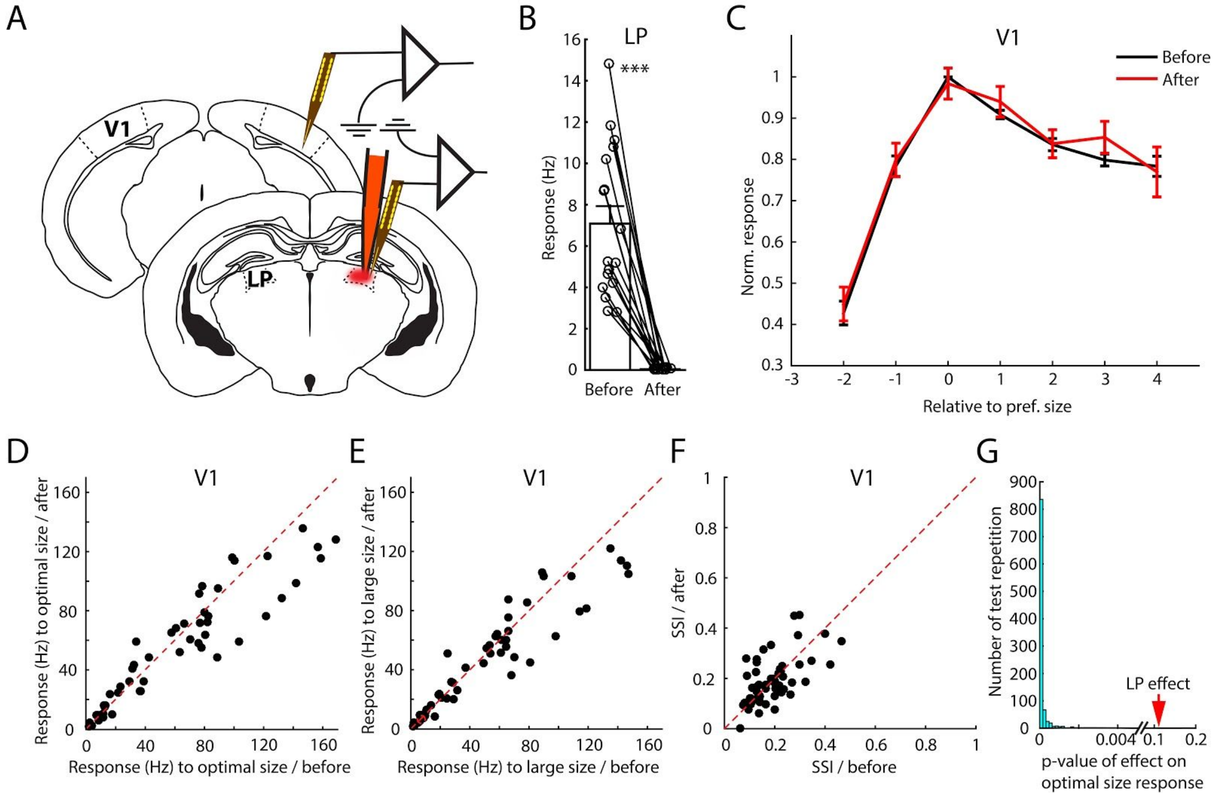

Supplementary Figure 4. No overall changes in V1 after silencing LP.

(A) Recording electrodes in LP and V1, and fluorescently-conjugated muscimol injected to LP. V1 electrode stayed at the same location before and after the muscimol injection.

(B) Response in LP before and after injection of muscimol in LP (4 mice, 17 units).

(C) Normalized population response in V1 to different sizes, relative to optimal size, before and after muscimol injection in LP (4 mice, 55 units). Error bars indicate mean ± s.e.m.

(D) Responses of V1 units to optimal size stimuli, before and after muscimol injection in LP ($p = 0.11$, Wilcoxon signed-rank test).

(E) Responses of V1 units to largest size stimuli, before and after muscimol injection in LP ($p = 0.24$).

(F) Surround suppression index of V1 units, before and after muscimol injection in LP ($p = 0.87$).

(G) Histogram of p-values of a Wilcoxon signed rank test on the effect on optimal size response for 1000 random samples of 55 units of all the units shown in Figure 1G. For all samples, the reduction of optimal size in V1 response by optogenetically inhibiting the sSC remains significant. Arrow indicates the p-value of the same test for LP silencing.

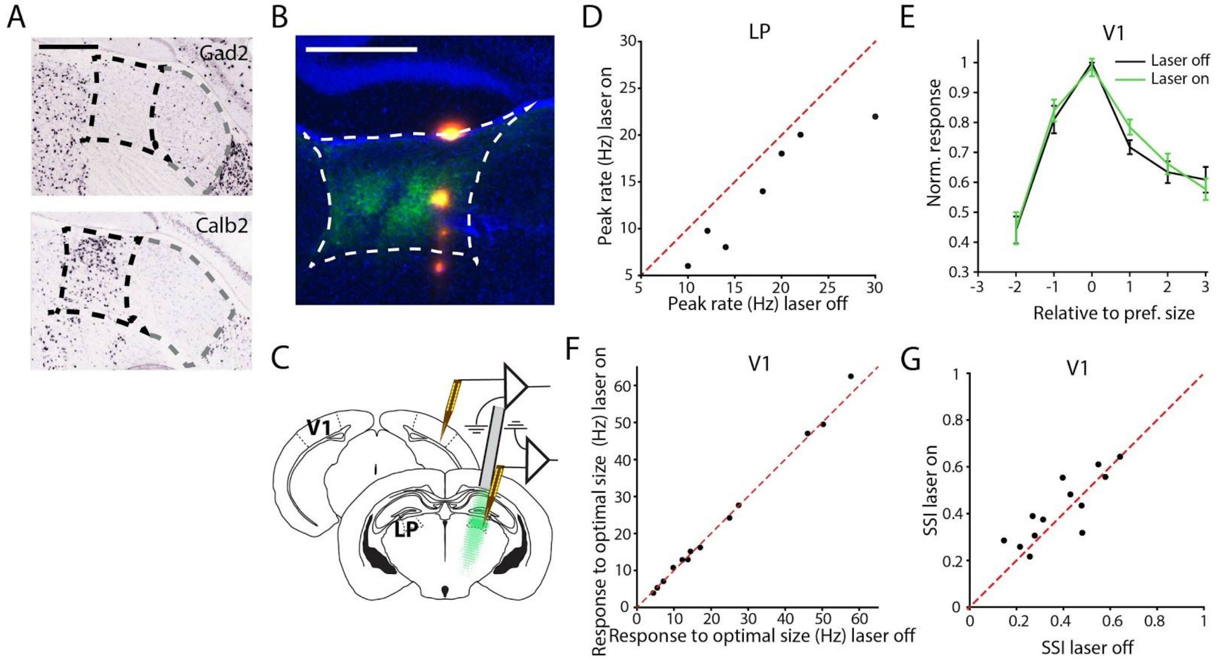

Supplementary Figure 5. Optogenetically reducing activity in LP did not change V1 surround suppression.

(A) Top, in situ hybridization of Gad2 mRNA shows that there are very few inhibitory neurons in LP. Bottom, in situ hybridization of Calb2 mRNA shows expression in LP and not in the neighboring dLGN. Dashed black and gray outlines mark LP and dLGN, respectively. Scale bar is 420 µm. Image credit: Allen Institute.

(B) Coronal slice through LP, showing Arch-GFP expression (green) in Calb2 neurons and the DiI trace left by the recording electrode (red/yellow). Scale bar is 0.5 mm.

(C) Recording electrodes in LP and V1 of anesthetized Calb2-Cre mice transfected with a Cre-dependent Arch-GFP expression vector in LP. A laser-coupled fiber was inserted into the brain, ending 1.5 mm above LP.

(D) Peak rate in LP in response to a full screen luminance increase was reduced when laser was turned on ($p = 0.003$, paired t-test; 2 mice, 7 units).

(E) No change in V1 population size tuning curve when LP was optogenetically inhibited. Error bars indicate mean ± s.e.m.

(F) No change in V1 response to optimal size visual stimuli when LP was optogenetically inhibited ($p = 0.84$, Mann-Whitney U test; 2 mice, 13 units).

(G) No change in V1 surround suppression when LP was optogenetically inhibited ($p = 0.22$, paired t-test; 2 mice, 13 units).

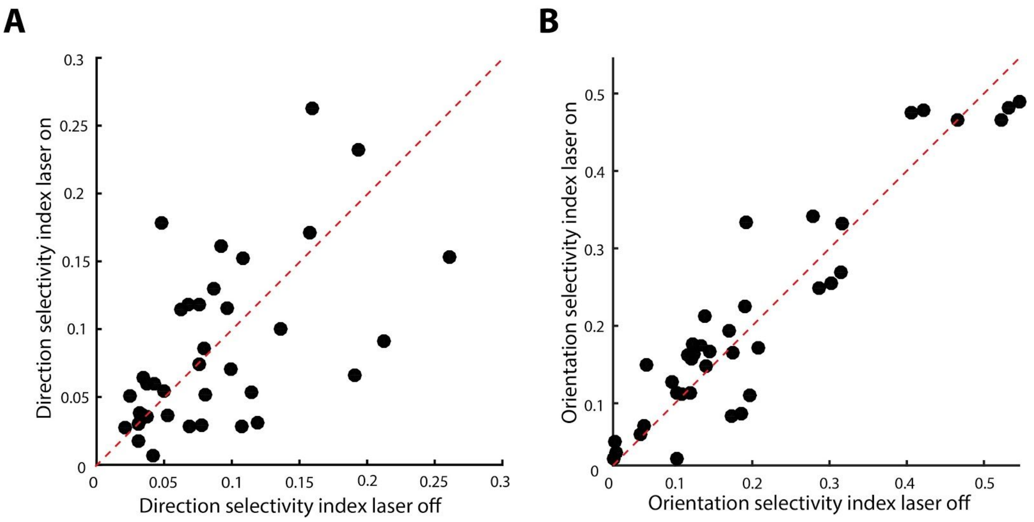

Supplementary Figure 6. Mean direction and orientation selectivity not changed by inhibiting sSC.

(A) Direction selectivity in V1 was not changed by optogenetically inhibiting sSC ($p = 0.89$, Wilcoxon signed rank test; 15 mice, 38 single-units).

(B) Orientation selectivity in V1 was not changed by optogenetically inhibiting sSC ($p = 0.39$, Wilcoxon signed rank test; 15 mice, 38 single-units)

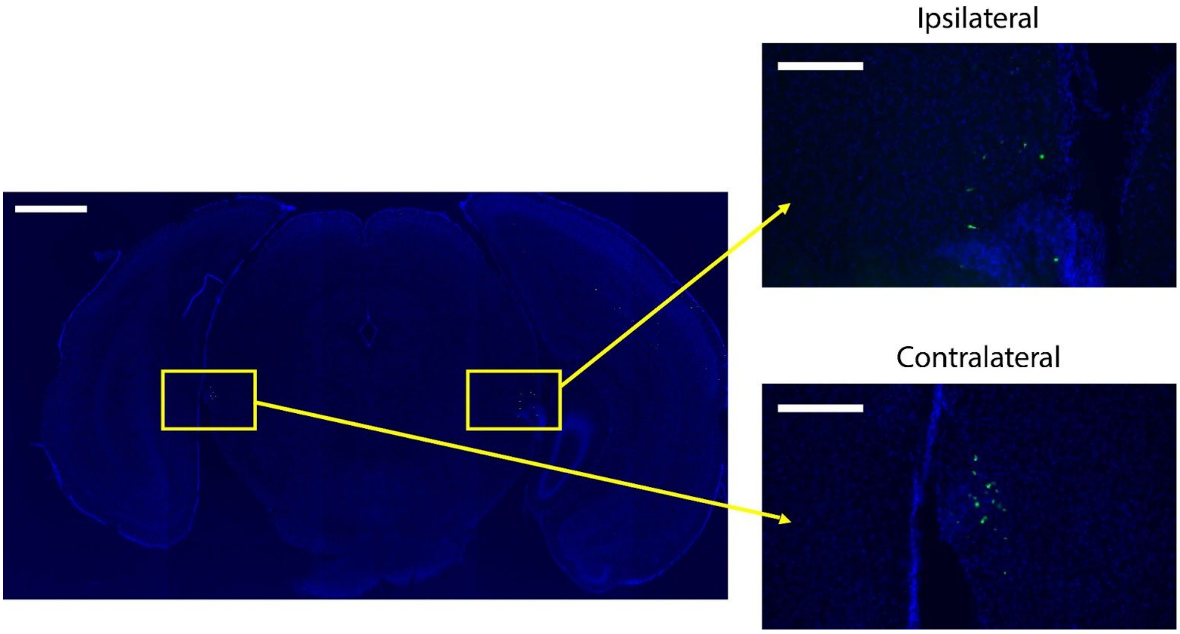

Supplementary Figure 7. Neurons in the ipsi- and contralateral PBG project to the dLGN.

Left. Coronal slice through the whole brain after a unilateral injection of the retrograde CAV2 virus expressing GFP (green) in the dLGN. DAPI in blue. Scale bar is 1 mm. Right top, magnified part showing labeled neurons (green) in the PBG ipsilateral to the injection site. Scale bar is 250 µm. Right bottom, magnified part showing labeled neuron in the PBG contralateral to the injection site.

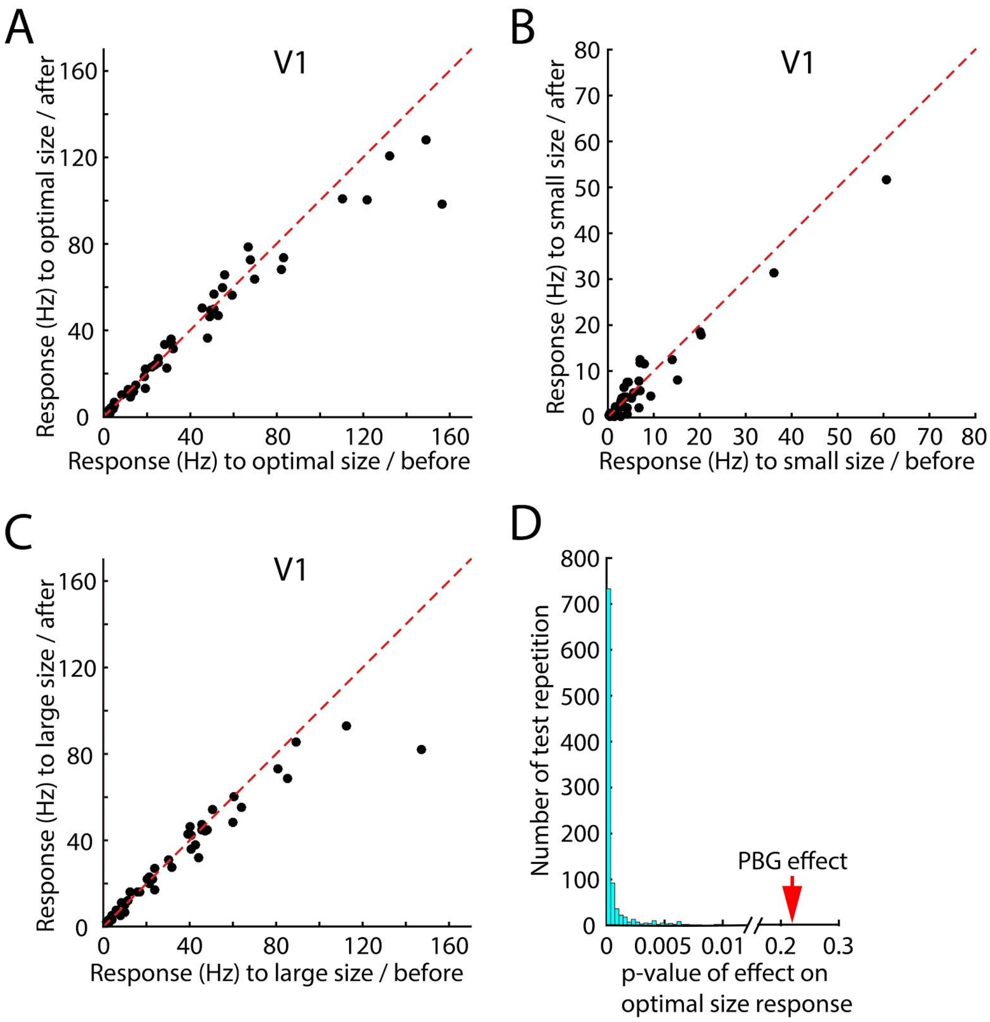

Supplementary Figure 8. Effect of PBG silencing on V1 responses.

(A) Responses of V1 units to optimal size stimuli, before and after silencing PBG by muscimol ($p = 0.22$, Wilcoxon signed rank test, 4 mice, 43 units).

(B) Responses of V1 units to the small size stimuli ($p = 0.10$).

(C) Responses of V1 units to the large size stimuli ($p = 0.12$).

(D) Histogram of p-values of a Wilcoxon signed rank test on the effect on optimal size response for 1000 random samples of 43 units of all the units shown in Figure 1G. For all samples, the reduction of optimal size in V1 response by optogenetically inhibiting the sSC remains significant. Arrow indicates the p-value of the same test for PBG silencing.
